# A Practitioner-Led Approach to Understanding Multi-Professional Working Across Health and Care: Co-Constructing the Integrated-Component Assessment Framework (I-CAF) and the Development Matrix for Integration (DMI)

**DOI:** 10.5334/ijic.9136

**Published:** 2026-03-18

**Authors:** Mark Llewellyn, Sophie Randall, Carolyn Wallace, Chiquita Cusens, Kerrie Phipps

**Affiliations:** 1 Welsh Institute for Health and Social Care, University of South Wales, Pontypridd, CF37 1DL, United Kingdom; 2 Strategic Programme for Primary Care, NHS Wales, United Kingdom

**Keywords:** practitioner-led team assessment, interprofessional and multi-professional working, health and social care integration, co-construction

## Abstract

**Introduction:**

This paper has two key purposes – to describe and introduce the Integrated-Component Assessment Framework (I-CAF) and the Development Matrix for Integration (DMI).

**Methods:**

The I-CAF is a new approach to practitioner-led team assessment, comprised of three methodological component parts – a Scoping Review, a Group Concept Mapping study, and Collaborative Action Research with practitioners. We then go on to describe and outline how the findings from those three components and the context within which the I-CAF was established have led to the co-construction of the DMI.

**Results/Discussion:**

The DMI is an assessment matrix for completion by a range of professionals engaged in interprofessional and multi-professional working across community health and social care services in both the public and voluntary sectors. Through utilisation of this new approach, a robust self-assessment tool is promoted which enables stakeholders to identify their team’s strengths and areas of development, and to reflect on change over time.

**Conclusion:**

The paper also identifies that the I-CAF approach may be relevant and useful to other areas of practice within and without health and social care. If adapted, the DMI could also provide a template for development in other areas of service.

## Introduction: multi-professional working in context

Multi-professional working is often viewed as a solution to challenges faced between professionals when working with people who are experiencing complex health and care problems [1]. The term multi-professional working is often used interchangeably with multi-disciplinary working and multi-agency working, all of which have previously been defined as a situation in which “staff with different professional backgrounds and training work together” [2,5].

The prioritisation of multi-professional working has been mirrored across health and social care systems, including in the UK by NHS England, who have stated that multi-professional working consistently delivers better outcomes for patients and more satisfaction among professionals [3], in addition to both NHS Scotland [4] and Health and Social Care Northern Ireland [5]. This is perhaps unsurprising given the links between high quality multi-professional working and improved patient outcomes [6,7], improved confidence of professionals [8], improved patient reach [9] and increased patient engagement [10,11].

Despite the benefits, multi-professional working can also present a host of challenges to contend with. Particularly following on from Covid-19 and the disruption to health systems [12], finding the time and budget to sufficiently facilitate multi-professional working can be difficult, hindering its success [13,14]. Similarly, high staff turnover may be a barrier to effective multi-professional working [15] as it hinders the ability of staff to form trusting relationships, instead giving rise to a lack of understanding and mistrust [15,16]. Challenges such as these limit the potential benefits of multi-professional working, ultimately to the detriment of the person accessing a service [17].

Within Wales, multi-professional working has been identified as a priority in order to bring about the integration of health and social care services by the Welsh Government [18]. Most recently, the Strategic Programme for Primary Care in Wales – an all-Wales NHS-led programme that works in collaboration with Welsh Government – published an agreed definition of multi-professional working which emphasises the culture and outputs expected from this workforce approach: “Multi-professional working describes a group of individuals across health, social care, independent and third sector, working together in a professional way as equal partners to ensure effective and smooth coordination in the delivery of person-centred care and support. Individuals working in this way may belong to separate professional groups, organisations, or different disciplines within a professional group” [19,4]. In this paper, ‘health’ relates to the range of services directly provided by or commissioned by the NHS in the UK; ‘social care’ relates to the a range of activities that include social work, personal care, protection or support to children or adults in need or at risk, directly provided by or commissioned by local authorities; and the ‘third sector’ relates to a number of non-governmental non-profit organisations that provide health and social care services, directly funded by government, or commissioned by the NHS and/or local authorities.

To mitigate some of these challenges and in alignment with the Welsh Government’s aspirations for multi-professional working, the Strategic Programme for Primary Care launched the Community Infrastructure Programme, the ambition of which is to “define the fundamental infrastructure required to deliver a place-based 24/7 integrated, multi-professional community model” [20]. A key question for the programme was how to understand what effective multi-professional working is, and how it could be measured over time.

## Background: Understanding effective multi-professional working

### Integrated care measurement

Two notable approaches have been developed in recent years to address this question of understanding and measurement: the Development Model for Integrated Care (DMIC) and the Scaling Integrated Care in Context project (SCIROCCO). Both have taken a structured and developmental approach to understanding how integrated care works in practice. Like the approach we describe below in this paper, the DMIC [21,22] was based on a systematic review of the literature surrounding integrated care [23] and augmented with a Delphi and concept mapping study [24]. The research team then engaged in a series of additional studies to validate their findings [25,26,27] which led them to an initial list of 89 elements of integrated care [21], which have recently been updated and renewed to a list of 108 elements [22], organised into nine clusters. These elements were brought together in a web-based self-assessment tool which can be used by integrated care services to help to understand the relevance and presence of each element in their integrated care practice. The tool asks about each of the elements, but uses a structured scale with which to do this. The SCIROCCO study took a similar approach undertaking a literature review and Delphi study to ensure that the B3-Maturity Model with its 12 dimensions was valid in measuring integrated care [28,29]. It does this by using six statements across each of the 12 indicators – respondents are asked to align their views with one of these statements each time.

What unites both of these approaches is that they have focused on ‘measuring’ integrated care. The approach of DMIC and SCIROCCO was to develop valid scales for measuring, benchmarking and comparing components and elements of integrated care. The approach is similar to the one described in this paper, but we were keen in our work to extend this. We have done this by providing 225 detailed qualitative ‘statements’ describing the experience of those working in integrated care settings in practice, using the Maturity Matrix idea, structured around eight key domains of activity. Our work is complementary to the DMIC and SCIROCCO, and as such the purpose of this study was to develop and describe a new way of thinking about how practitioner-led self-assessment approaches can be developed; and to co-construct a self-assessment matrix for those undertaking multi-professional working in the community, as below.

### Maturity Matrices and the approach to devising them

Originally designed by Elwyn et al [30] as a way to assess organisational development in primary medical care, the Maturity Matrix was first promoted as an effective way to engage professionals at all levels of a multi-disciplinary team. The Maturity Matrix ensured that a range of voices were accounted for whilst not requiring a large time commitment to complete. Implementation of the Maturity Matrix was a success, and as a result Maturity Matrices are now in use across many facets of health and social care, and in many other businesses and across the public sector – like within genomics [31], psychiatric care [32], within NHS England’s integrated care systems [33], community pharmacy [34], or in support of large-scale changes in health systems [35]. Part of the success of the Maturity Matrix can be attributed to its high face validity and feasibility for use in practice [36], meaning that for almost twenty years such matrices have been viewed as valuable and continue to be used in some of the most recent research examining care [37].

Despite such use, studies that employ a Maturity Matrix appear to lack consensus on the most effective means by which they are devised. For example, the matrix developed by Wolters et al [37] is informed by a scoping review, something that is not present in other studies. Similarly, Lewing et al [38] include accompanying information such as a table to guide users on ‘what good looks like’, something missing from other recent matrices. Tonkin et al [31] used intensive three-day workshops to inform their matrix, garnering a high level of professional consensus through electronic voting, whereas Black and Luming [32] describe how their matrix was developed during monthly organisational meetings exclusively attended by medical directors and quality leaders – an approach similar to that of Teichert et al. [34]. Therefore, despite the umbrella term of ‘Maturity Matrix’ applying to each of these studies, there are differences in the methods used to produce matrices, and in the form that the outputs take as a result.

Within this paper, we outline the way in which we have co-constructed – alongside the clinical leads within a national programme for primary care, and practitioners working across community health and social care – a new approach to thinking about how to design a Maturity Matrix, placing it alongside other component parts to develop a self-assessment tool for multi-professional working in the community. The Integrated-Component Assessment Framework (I-CAF) builds on the learning from the production of previous Maturity Matrices to embed its own matrix into one cohesive framework, using a Scoping Review, a consensus-based Group Concept Mapping study, and co-constructed collaborative action research conversations to ‘bake in’ the views and experiences of practitioners to inform our final matrix – the Development Matrix for Integration (DMI). The DMI allows practitioners to understand their team’s approach to multi-professional working across health and care, so named as it identifies clear areas for development and improvement post-assessment.

As indicated, the work for the Strategic Programme for Primary Care required us to think about the development of a self-assessment toolkit for multi-professional working in the community with similar features and benefits to Maturity Matrices (i.e. non-time consuming, able to give voice to all members of a team). However, given the differences between past matrices and some of the gaps identified in addition to the sensitive nature of developing such a matrix for multi-professional working, it was felt that a new methodological approach would be needed, and so the I-CAF was first conceptualised with the DMI as the key output from three component methods (detailed below).

To learn from previous work, the I-CAF and resulting DMI needed to build on the lessons and guidance of past studies, for example by incorporating good practice examples such as was done by Lewing et al [38], building consensus akin to Kirk et al [39], gathering perspectives through practitioner-led discussions [40,41] to take a highly participatory approach to ensure validity similar to Tonkin et al [31]. Furthermore, the I-CAF would extend these approaches by providing a strong evidence-base at the outset which could be referred back to throughout the process, ensuring that the final output had additional validity in being able to build on the published literature.

## Method: Rethinking the Maturity Matrix – towards an integrated approach

As such, the three key component methods of the I-CAF (leading to the resulting DMI being produced) were determined as follows:

1. A Scoping Review to map the evidence-base and describe the extant literature;
2. A Group Concept Mapping study to established consensus from practitioners on the most important, impactful, and easy to collect data; and
3. Collaborative action research with practitioners [40] to co-construct the wording of domains, indicators and descriptors that would be used within the DMI.

**Figure 1:**
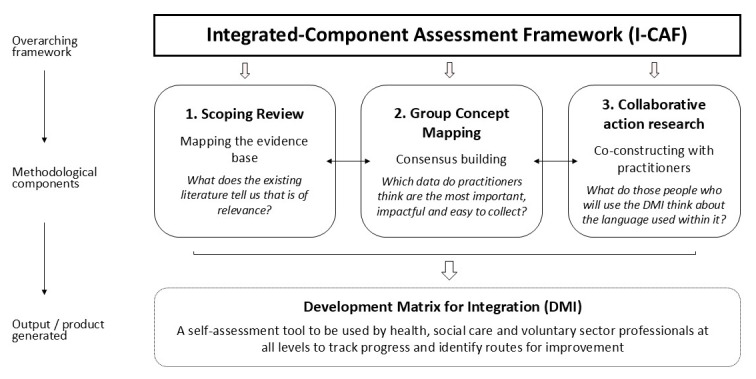
The Integrated-Component Assessment Framework (I-CAF).

As shown in Figure 1, taken together, these three components (which led to the key output of the DMI) form the I-CAF. Although each component followed its own process, method and approach, they were integrated so that each built upon the others to form the final cohesive Framework.

In the sections that follow, we describe the new approach to the co-construction of the DMI through the three components. As such this provides a ‘worked example’ of how the approach can be applied to multi-professional working across health and social care, but it is important to note that the I-CAF approach could equally be applied to other contexts. We use the phrase ‘practitioner-led’ throughout this paper to refer to two processes – the way in which the collaborative action research was indeed led by practitioners’ views, but also that the final DMI is designed to be practitioner-led, and does not require the self-assessment to be facilitated by others. In the following section of ‘Results and Findings’ we also provide some additional detail on the methods and approach used.

## Results and Findings

### I-CAF Component 1: the Scoping Review and forming an evidence base

The first component to be undertaken was the Scoping Review [42] to map the existing evidence base around multi-professional working in the community. This was carried out between June–August 2022 by the research team (i.e. the authors of this paper) and aimed to answer six core questions:

1. What literature is there which describes multi-professional working in the community?
2. What workforce models are in the examples identified in the literature?
3. What benefits are there to multi-professional working in the community?
4. What literature is there which describes the patient experience of multi-professional working in the community?
5. What are the challenges of multi-professional working in the community?
6. What literature is there which explores the health economics of multi-professional working in the community?

A PICO (Population/Problem, Intervention, Comparison, Outcome) table was developed to assist in generating search terms for databases. Terms were limited to those closely aligned to the research questions and therefore synonymous with the terms ‘multi-professional’, ‘community’, ‘health economic’ and ‘model’. Four databases were searched with terms kept consistent throughout and only adapted (i.e., to add an additional term) if too large a sample of literature was returned for review. A total of 567 records were identified through database searches, 210 duplicates were removed leaving 357 records for screening. Records were first screened by both titles and abstract by members of the team.

Following title and abstract screening, a total of 112 records were included. Of these, nine could not be retrieved in full, 15 were study proposals without posted results, 13 were not contextually relevant when read in full and one had been referenced as part of another review, leaving a total of 74 papers in the final review.

The review answered all six of its research questions and identified key values, benefits and challenges of multi-professional working, which are summarised in Table 1. Overall, there was no consensus on a singular definition of multi-professional working nor an agreed upon workforce model for success, though co-location was mentioned most frequently. Patient experience of multi-professional working was largely demonstrated to be positive when multi-professional working is carried out effectively and the literature also suggested a range of health economic benefits to multi-professional working when it was properly supported.

**Table 1:** Key values, benefits, and challenges of multi-professional working as identified by the scoping review.


VALUES	BENEFITS	CHALLENGES

Early intervention	Cost-effectiveness	Budgetary restrictions

Trust	Improved outcomes	Staff turnover

Communication	Patient satisfaction	Lack of understanding

Collaboration	Knowledge sharing	Poor communication

Equity	Faster assessments	Mistrust

Empowerment	Improved patient reach	Access to records

Quality improvement	Increased engagement	Differing priorities

Innovation	Fewer admissions	Poorly defined responsibility

Accessibility	Shorter hospital stays	Time restraints

Sustainability	Fewer crisis referrals	‘Territorial’ behaviour


In completing the Scoping Review, a range of issues were surfaced that were then taken forward to inform the subsequent components of the I-CAF. This initial evidence-base was drawn upon and referred back to in both the Group Concept Mapping study and the DMI, using it to sense check other results and to ensure that the final product would remain aligned with the extant literature.

### I-CAF Component 2: Group Concept Mapping to gain consensus

Between July-November 2022, the Group Concept Mapping [43] component was carried out. Ethical approval for this study was sought and given by our institutional ethics committee (220601LR). As required by Health and Care Research Wales processes, permission to include NHS staff was granted by each participating NHS organisation. All contributors consented to their participation in the study.

Group Concept Mapping is a structured methodology used to generate consensus among a diverse group of stakeholders [44]. It systematically integrates qualitative components of a study through the processes of idea generation, with quantitative components through the representation of those ideas in visual maps and other reports. These may then be used to generate a conceptual framework for the subject of inquiry [e.g. 45,46].

As a form of online consensus building, the purpose of the Group Concept Mapping component of the I-CAF was to derive areas of agreement on how to measure multi-professional working. It did this through understanding the views of those who work in multi-professional teams on what to measure in the context of multi-professional working in the community, and how such data could be collected. To this end, the Group Concept Mapping exercise sought response to four key questions:

1. What data should be included within the I-CAF?
2. What do professionals think is the most important data?
3. What do professionals think is the most impactful data?
4. What data do professionals consider easiest to collect?

Key themes were generated from this work in addition to specific data items ranked across domains. There are three key activities of the Group Concept Mapping method: ‘brainstorming’ statements, sifting and ‘sorting’ statements into themes with labels, and ‘rating’ each statement for importance, impact, and ease of collection. Participants were drawn from the existing networks of the Strategic Programme for Primary Care, and different numbers of respondents took part in the three key activities, which is typical for Group Concept Mapping studies [for more, see reference 43].

The ‘brainstorming’ phase of Group Concept Mapping invites participants to generate their own statements to a ‘focus prompt’. The generation of these statements is key to the process, and during this activity, participants (*n* = 15) together provided 87 statements to complete the single online focus prompt: *“When developing a multi-professional working framework, I think I would measure the value of this way of working as…”*. These 87 statements were split where there was more than one data item included in a response, giving 161 statements generated by participants in total. In addition to these, 21 statements were added to the list from the content of a previous study which considered a commensurate area of multi-agency working [47]. Finally, the researchers reviewed the statements against the findings from the previous component of the I-CAF, the Scoping Review [42] to ensure that the issues raised in those papers had been covered by the responses of participants. To a very large extent they had been, but six statements were added from the results of the Scoping Review. This left a total of 188 statements before ‘cleaning’ (i.e., duplicates removed, wording altered for clarity). Following cleaning, 123 statements were included for sorting and rating, of which 100 were generated by participants, 17 were included from a previous study, and 6 were generated from the results of the Scoping Review.

In the ‘sorting’ activity that followed, participants (*n* = 42) were asked to group all statements into piles (which is done through the software online) and provide each pile with an individual label. This provided first a ‘point map’ in which all 123 statements were mapped, with the distance between points representing how often two statements had been sorted into the same pile. The software then provided a cluster map where statements were sorted as in the point map, but also distributed across five clusters or ‘themes’.

Finally, participants were asked to rate all 123 statements on 5-point Likert-type scales for the importance of that statement for understanding multi-professional working (*n* = 40), the impactfulness that the statement would have for comprehending multi-professional working (*n* = 36), and ease of collecting those data items (*n* = 37). Overall, “patient/person centred” was considered by participants to be the most important and impactful cluster of statements, and “workforce and staff wellbeing” was seen as the easiest to collect. Individual statement ratings were also examined and the top five statements across each domain in addition to the top five statements collectively were identified (Table 2). The column labelled ‘Combined’ is a list of the top five statements from the other columns together.

**Table 2:** Top five ranked statements by participants in each domain.


	IMPORTANCE	IMPACT	EASE OF COLLECTION	COMBINED

1	Delivery of safe, high quality, effective care	Delivery of safe, high quality, effective care	Staff retention	Staff retention

2	Person receiving timely, coordinated, collaborative care	Person receiving timely, coordinated, collaborative care	Regular multi-professional meetings	Staff satisfaction

3	Respect for others	Staff wellbeing	Staff satisfaction	Using shared IT systems

4	Valuing the peoples’ voice	Maximising people’s independence	Using shared IT systems	Staff wellbeing

5	Enabling people to have involvement in their care	Positive leadership	Co-location of services	Measuring service user perspective


The top five most important, impactful, and easy to collect statements could be considered as hierarchical priority statements in regard to the data that professionals engaging in multi-professional working value, and important to the I-CAF in this context. Undertaking this Group Concept Mapping provided an additional evidential underpinning for the I-CAF and ensured that the consensus of local professionals was taken into account in its further development.

### I-CAF Component 3: Collaborative action research and co-construction with practitioners

In tandem with finalising the Scoping Review and conducting the Group Concept Mapping study, the co-construction of the DMI began. Key to this was utilising the method of collaborative action research with practitioners to understand their experiences of multi-professional working [40].

From the outset (between August and October 2022), the team engaged practitioners in a series of meetings to discuss the matrix in the project’s context of multi-professional working more widely. Professionals came from a range of health, social care, and third sector settings. These ‘collaborative conversations’ [40,41] were not formal interviews or focus groups and so allowed for free-flowing discussion with practitioners in which they were able to give the team an honest reflection of how they were currently engaging with multi-professional working, what they felt the key successes and barriers to multi-professional working in their role were, and how they understood their ‘relational agency’, their capacity for working with others to strengthen purposeful responses to complex problems [48]. In total there were 15 such meetings – some were on a one-to-one basis, others were with small groups. In total 20 contributors provided input across these 15 meetings. These contributors were drawn from the health, social care and third sector, and from various service areas which included GP clusters, social prescribing, community resource teams, physiotherapy, community connection, dietetics, optometry, community pharmacy, occupational therapy and ambulance services.

Using the notes made in these conversations, key themes were identified and were then triangulated with the findings of the Scoping Review and the Group Concept Mapping, which provided a rich and evidence-based underpinning for the perspectives given by professionals. Through integrating these components, high level and overarching themes concerning multi-professional working were identified in addition to a series of sub-themes – these would become the domains and indicators of the first draft of the DMI. This method allowed for the research team to develop an initial understanding of the emergent component parts of the DMI.

Once the first draft was completed, a second round of collaborative action research conversations were held with professionals between January and March 2023. These discussions sense-checked the DMI, informally testing it for face validity, and provided suggestions for additional domains or indicators and any changes to the wording of statements. In effect, these practitioners tested and evaluated the usability of the matrix in practice and made suggestions back to the research team for how it could be improved. These included concerns over the length of the DMI and its usability, which led to a determination that the different domains should be constructed as ‘stand-alone’ sections. In this second ‘round’, 21 such conversations were held with 33 professionals who completed 15 self-assessments overall. Following these conversations, the DMI was redrafted to address any comments made and the top-ranking statements from the Group Concept Mapping study (which themselves had been triangulated against the Scoping Review) were integrated within the main body of the DMI as suggestions for data that users may would want to collect or consider.

In March 2023, a Community of Practice (CoP) was established with those who had been engaged in co-constructing the DMI to date, and augmented by those who had an interest in its future development drawn from the existing networks of the Strategic Programme for Primary Care. Once again, attendees had backgrounds across health, social care, and the third sector. This method was selected in order to provide additional opportunity for challenge and critique from a broader group of practitioners. CoP meetings were led by the research team (i.e. the authors of this paper).

The first CoP meeting focussed on one key question in particular (How do we better collaborate?) with a view to establishing a series of options that would allow those who had completed a self-assessment using the DMI to share areas of strength, and areas of development with others. The CoP engaged in a rich and detailed discussion during which attendees discussed both enablers of, and barriers to, multi-professional working and reflected on their experiences using the DMI so far. Following this CoP, the research team undertook a series of tasks ahead of the second meeting, in particular as practitioners had asked for links to be made throughout the DMI to good practice examples that could be referred to when completing a self-assessment, so that there would be an idea of ‘what good looks like’. This work was undertaken with examples being extracted from the Scoping Review, from reputable resources such as the King’s Fund and Nuffield Trust, and from suggestions provided by professionals themselves.

A second CoP was held in May 2023, this time focussed on the newly re-drafted DMI. Attendees had opportunity to provide any further feedback on the DMI in addition to providing comment on ways it might be disseminated or integrated into workforce planning. There was a clear consensus during this CoP that the professionals who attended were satisfied that the DMI represented multi-professional working within the community well and no substantive changes were suggested. A final CoP was held in June 2023 in which the DMI was fully agreed upon by attendees for use in practice.

## Discussion: Output from the I-CAF – the Development Matrix for Integration (DMI)

Aligning the evidence gathered from the three Components, the finalised output – the DMI – provides a means by which a practitioner-led qualitative assessment can be made of progress towards effective multi-professional working by the engaged in such work.

The DMI is designed so that teams and services can determine which of the descriptors in the cells in the matrix best describes their progress to date against different dimensions within eight domains. An overview of the eight domains and 45 indicators of the DMI is provided in Table 3.

**Table 3:** An overview of the eight domains and 45 indicators included in the DMI.


DOMAIN	INDICATORS

1. Buy-in	1.1 | Connections with people, patients and the wider public

	1.2 | Strategic and senior leader engagement

	1.3 | Influence within key organisations (health board and/or local authority and/or third sector) processes

	1.4 | Buy-in of other professionals

2. Internal relationships, collaboration, and cohesion	2.1 | Internal leadership and culture

	2.2 | Shared vision, common direction, culture and purpose

	2.3 | Opportunities for meeting and discussion

	2.4 | Networking with others

	2.5 | Communication between professionals

3. External relationships	3.1 | Engagement with social services

	3.2 | Engagement with the third sector

	3.3 | Engagement with secondary care/community hospitals

	3.4 | Engagement with primary care

	3.5 | Engagement with other key stakeholders [only if additional and relevant to 3.1, 3.2, 3.3, 3.4]

4. Information sharing and governance	4.1 | Appropriate IT systems access

	4.2 | Communication between IT systems

	4.3 | Record keeping

	4.4 | Confidentiality and consent

	4.5 | Clear policies and procedures

	4.6 | Robust systems for information governance

5. Equity and equality	5.1 | Geographical equity

	5.2 | Managing capacity

	5.3 | Parity of esteem

	5.4 | Voice of those closest to the person

6. Person-centred practice	6.1 | Focus and responsivity of the service

	6.2 | Flexibility and responsivity of the practitioner

	6.3 | Clear definition of professional roles

	6.4 | Feedback from people supported by the service

	6.5 | Clarifying what matters to the person the service is supporting

	6.6 | Involvement and communication with a person’s support network (where appropriate to the needs of the person)

	6.7 | Communication and language preferences

	6.8 | Flows through the whole system

7. Resources – human and financial	7.1 | Staff retention

	7.2 | Staff recruitment

	7.3 | Budget

	7.4 | Development of individual team

	7.5 | Individual staff development

	7.6 | Space for physical co-location

	7.7 | Virtual co-location

8. Embedding data in practice	8.1 | Culture of quality improvement and learning

	8.2 | Appropriate range of skills and systems

	8.3 | Quality of data and evidence

	8.4 | Embedding data in processes of improvement

	8.5 | Shared learning between teams

	8.6 | Co-production of service and quality improvement


This is not the full DMI – the matrix in its entirety is supplied as a Supplementary File to this paper. The full version of the DMI contains five descriptors for each indicator moving from the earliest form of multi-professional working at ‘Statement 1’ through to an optimised form of practice at ‘Statement 5’. Through the three components of the I-CAF, the DMI has been co-constructed to allow services and teams to compare their progress over time, and also to allow for a conversation between services working in different multi-professional contexts, primarily within Wales.

Using the domains, indicators and statements enables that conversation to take place, such that teams can identify areas of strength and development, share those with others, and engage in a dialogue about why there might be differences in their scores. In addition to the domains, indicators and statements included in the DMI, The King’s Fund [49] published a ‘Reflective Learning Framework for Partnership’ drawn from their work on integrated care partnerships. This has been integrated into the DMI as a series of ‘test questions’ appearing throughout the matrix, intended to aid reflection for users ahead of making their self-assessment of the team’s effectiveness.

Along the top of each domain are suggested data relating to the way that domain might be measured or improved. These were identified through the Group Concept Mapping study (I-CAF Component 2) are the top 30 ‘most important and impactful data items’ according to the professionals who took part in that study. These data are only intended to act as suggestions, however they do represent the consensus within the participants of the most important and impactful items.

Using the DMI to identify areas for service and/or team and/or individual development is the key purpose of the DMI. Accordingly, there is an underlying logic in how the statements within the matrix build on one another. The statements are incremental; inherent in moving along from one statement to another is the assumption that forms of practice under the previous statement are implied within the next. However, it was clear in our engagement with practitioners that due to variations in what they do, and the hours that they work within (whether daytime, or 24/7), not every indicator within each domain will be relevant to every setting. Accordingly, there is an opportunity to provide context around the statements in the box underneath the matrix. There is also an ‘N/A’ option which can be used if the domain/indicator is determined to be outside of the current remit of the service model, is not required in that locality, or if there is capacity elsewhere in the system that can be drawn upon.

Therefore, the intention of the DMI is for individuals and/or teams to make a self-assessment of their team’s effectiveness against each of the domains and indicators that are relevant to them. It is the detail of the descriptions that accompany these indicators (five qualitative statements for each of the 45 indicators has been drafted) that differentiates the DMI from previous tools, enabling users to clearly identify areas for development. Users of the matrix are encouraged to fill in the matrix using a darker shading to denote where there is greater evidence that a statement has been fully achieved, or a lighter shading to identify where some progress has been made in a domain, but that it remains a ‘work in progress’.

It is important to note that the matrix can be deployed variously within different contexts. There could, of course, be one ‘composite’ matrix that is completed at service/multi-professional team level, and this single matrix can be an amalgamation of a number of different matrices that have been completed by operational teams, managers, stakeholders and others – or indeed it can be used as a single stand-alone assessment. It is useful also to reflect on the purpose for completing the matrix – whether it is for reporting, and/or for evaluation, and/or for learning. These are not mutually exclusive of course, but it is worth being clear for those completing the matrix as to why they are doing so. However, it should be used longitudinally, with assessments typically made in cycles of 6 to 12 months to determine ‘distance travelled’ over time.

Finally, whilst the DMI has a current ‘final’ form, this is likely to change. As new priorities and initiatives emerge across health, social care, and the voluntary sector, the role of multi-professional working is also likely to change, and at such time the DMI will be adapted accordingly. It is also the case that as the DMI is used by practitioners, inevitably there will be additional suggestions as to how it can be amended and improved – the DMI is an iterative document and will remain so. Any changes or amendments to the DMI will be taken forward in the same co-constructive and robust manner as its original inception.

There are of course limitations associated with the approach outlined in this paper. The collaborative action research process was a powerful way to develop the tool but, as with any such process, we needed to be aware of power dynamics in the conversations – both in terms of the seniority of some staff, but also between different sectors – health, social care and the third sector. The I-CAF and DMI as described in this paper were developed within the context of interprofessional and multi-professional working in Wales, and so are primarily designed for that purpose. However, the I-CAF approach is potentially applicable in myriad contexts. The findings of the scoping review is applicable to contexts outside of Wales, and whilst drawn from Welsh health and social care professionals, the other methodological components underpinning the I-CAF could be replicated in any service and policy context to develop a similar integrated framework for any type of setting. As such we advocate that the DMI is generalisable across health, social care, and voluntary sector contexts elsewhere within the UK and internationally – it may however need to be reviewed in other contexts to sure the degree of fit with those circumstances. It is currently being used in Wales and is hosted by the Strategic Programme for Primary Care as a resource which can be deployed to support better multi-professional working.

## Conclusions

The aim of our work, and by extension of this paper, was two-fold: to develop and describe a new method for thinking about the ways in which practitioner-led self-assessment approaches can be developed – the I-CAF; and to specify, iterate and co-construct a self-assessment matrix for professionals engaged in multi-professional working in the community – the DMI. To use the I-CAF is to undertake and integrate three method components. Firstly, to conduct a Scoping Review with a question of relevance at the heart of the process. The findings of the review are then included in the second phase, to carry out a Group Concept Mapping study to build areas of consensus around the issue. Thirdly, to engage practitioners in collaborative action research conversations in order that the previous two phases are grounded in the realities of working practices. These three methods build one on another and, when integrated, allow for the co-construction of a Development Matrix, in this case focused on Integration and multi-professional working.

The DMI itself constitutes a comprehensive toolkit that is ready for use in practice, which has been co-constructed to:

- Describe what is happening with high face validity for key stakeholders.
- Facilitate a description of what is happening in a way that enable discussion between stakeholders.
- Illustrate what ‘good’ looks like, with steps to suggest and/or demonstrate development.
- Enable stakeholders to discuss amongst and for themselves how they perceive their current circumstances and agree on the next steps, as this is how such tools work best.

The descriptions of the approach taken provides guidance for others wishing to develop such a framework to do so in a way that is evidence-based, holds high face validity, and is potentially generalisable across a variety of service contexts.

## Additional File

The additional file for this article can be found as follows:

10.5334/ijic.9136.s1 Supplementary File. The Development Matrix for Integration (DMI) is the self-assessment tool that aims to support multi-professional working across groups.
