# Supplementary material for: A Practitioner-Led Approach to Understanding Multi-Professional Working Across Health and Care: Co-Constructing the Integrated-Component Assessment Framework (I-CAF) and the Development Matrix for Integration (DMI)
Source: Int J Integr Care. 2026 Mar 18;26(1):10. doi: 10.5334/ijic.9136 (PMC13004068; doi:10.5334/ijic.9136)
Supplement: Supplementary File. — The Development Matrix for Integration (DMI) is the self-assessment tool that aims to support multi-professional working across groups. [file ijic-26-1-9136-s1.pdf]

DEVELOPMENT MATRIX FOR INTEGRATION (DMI)

PURPOSE OF THE MATRIX

The Development Matrix for Integration (DMI) provides a framework for a qualitative assessment to be made of progress. The DM is designed so that services can determine which of the descriptors in the cells in the matrix best describes their progress to date against different dimensions within eight domains. It has been designed after conversations with 13 different services working in a multi-professional context across Wales. Accordingly it is important to note that not every single indicator of the 45 contained herein will be relevant to everyone (hence the 'N/A' option), and that in designing the DM we have ranged across a wide variety of service contexts.

It is intended that the DM helps services to compare their progress over time, and also to potentially facilitate a conversation between services in Wales working in a multi-professional context. One of the benefits of the DM is that services can use it to assess their progress in relatively short order – services do not need to engage in a detailed data collection exercise before they make a determination of progress against the matrix.

It is also important to note that it is a tool designed to support the development of multi-professional working (M-PW) – it is not a performance management tool, and it will only be useful if people are honest about where they feel their service is when an assessment is made. It is not just about getting to 'Statement 5' without thinking carefully about the stages before, and equally there is no problem in recognising that you may be at 'Statement 1'. We are concerned to reflect on 'development' in the matrix – where you start the journey is just an honest reflection on where things are.

Our aspiration in developing a matrix was to produce a tool that stakeholders – whether leaders, managers, practitioners and others – would find helpful. It was designed to:

- Describe what is happening with 'face validity' for the key stakeholders;
- Facilitate a description of what is happening in a way that enables discussion between stakeholders;
- Illustrate what 'good' looks like, with steps to suggest and/or demonstrate development; and
- Enable stakeholders to discuss *amongst and for themselves* how they perceive their current circumstances and agree on the next steps to be taken, as this is how such tools work best.

More information about the DMI, and a downloadable version can be found here: Multi-Professional Framework for Integrated Working - Primary Care One (nhs.wales)

Corresponding author:

Professor Mark Llewellyn

Director, Welsh Institute for Health and Social Care, University of South Wales, Pontypridd, CF37 1DL.

mark.llewellyn@southwales.ac.uk

Domain	S1	S2	S3	S4	S5	N/A
	**Descriptors: For each of the dimensions below, which statement below (S1-S5) best describes your current position?** *It is important to note that there is an underlying logic in how the statements build on one another across the matrix. The statements are broadly incremental – **moving along the boxes presupposes that forms of practice under the previous statement are included in the next one**.* *Darker shading against statements indicates that there is evidence that the statement has been fully achieved. Lighter shading is an indication that some progress has been made in this domain, but that it remains a 'work in progress'.*					
**1. BUY-IN** (Link to relevant film on SPPC channel: Domain 1)						
**Most important & impactful data items:** one system that supports people by putting them at the centre; agreed processes that enable seamless delivery between teams and services; co-ordinated service provision; strong leadership						
**1.1 | Connections with patients and the wider public**	Very limited public awareness of multi-professional working (M-PW) – there may be a view that this is cumbersome or unnecessary	Some public awareness of M-PW but this may not be fully understood or optimised	Increased public awareness and understanding of M-PW however many people may not be benefitting directly	Good public awareness and understanding of M-PW and its benefits – views are generally positive	Public awareness and understanding of M-PW and its benefits leads to impacts on services delivered	
**1.2 | Strategic and senior leader engagement**	Senior leaders are unaware of the work of the service and act in isolation without meaningful recourse to the work of the team	Senior leaders have some awareness of the work of the multi-professional team (M-PT) and may involve some team members in a limited capacity	Senior leaders have a good awareness of the work of the M-PT and usually involve the team in decision making	Senior leaders have a comprehensive understanding of the M-PT and always consider and involve the team	Senior leaders are actively engaged in the M-PT, understanding its impact and championing its value in collaboration with the team	
**1.3 | Influence within key organisations (health board and/or local authority and/or third sector) processes**	Lack of influence within the key organisations – M-PT only follows what others are already doing	M-PT begins to shape its identity albeit limited, and identify areas it would like to influence in future	M-PT has some distinct policies however has not fostered its own identity and has some input to organisational processes	Formation of distinct M-PT identity that begins to improve influence, status and standing	Ownership over identity and ability to effectively influence organisational processes and policies because of this	
**1.4 | Buy-in of other professionals**	Other professionals may lack understanding of the value of M-PW and are not invested in the process	Other professionals have some, but limited understanding of the value of M-PW, and become engaged in M-PW	Other professionals have a comprehensive understanding of M-PW and have a keen interest in the process	Other professionals begin to actively invest and engage in M-PW as they have a comprehensive understanding of its benefits	Other professionals are actively invested and engaged in M-PW and are advocates for its continuation	

<table>
<tr><td rowspan="2">Domain</td><td colspan="6">Descriptors: For each of the dimensions below, which statement below (S1-S5) best describes your current position?
It is important to note that there is an underlying logic in how the statements build on one another across the matrix. The statements are broadly incremental – moving along the boxes presupposes that forms of practice under the previous statement are included in the next one.
Darker shading against statements indicates that there is evidence that the statement has been fully achieved. Lighter shading is an indication that some progress has been made in this domain, but that it remains a ‘work in progress’.</td></tr>
<tr><td>S1</td><td>S2</td><td>S3</td><td>S4</td><td>S5</td><td>N/A</td></tr>
<tr><td>TEST QUESTIONS FOR DOMAIN 1
(from RLFfP, Q1)</td><td colspan="6">WHAT IS THE PURPOSE OF THE PARTNERSHIP’S WORK?
▪ Who are the intended beneficiaries of your work together?
▪ What impact does your partnership want to have?
▪ Who are you trying to influence?
▪ How are you collaborating to develop your partnership’s purpose?
▪ When will you need to articulate the partnership’s vision and who needs to be involved in this?</td></tr>
<tr><td>Useful resources and worked examples:</td><td colspan="6">Models and Frameworks for Coordinating Community Responses During Covid-19
Integrating health and social care (nuffieldtrust.org.uk)
Integrating primary care and social services for older adults with multimorbidity: a qualitative study (bjgp.org)</td></tr>
</table>

Domain	Descriptors: For each of the dimensions below, which statement below (S1-S5) best describes your current position?<br>*It is important to note that there is an underlying logic in how the statements build on one another across the matrix. The statements are broadly incremental – **moving along the boxes presupposes that forms of practice under the previous statement are included in the next one**.*<br>*Darker shading against statements indicates that there is evidence that the statement has been fully achieved. Lighter shading is an indication that some progress has been made in this domain, but that it remains a 'work in progress'.*					
	S1	*S2*	*S3*	*S4*	*S5*	*N/A*
**2. INTERNAL RELATIONSHIPS, COLLABORATION AND COHESION** (Link to relevant film on SPPC channel: Domain 2)						
**Most important & impactful data items:** supportive team climate; positive leadership; respect for others; shared vision; delivery of safe, high quality, effective care						
**2.1 | Internal leadership and culture**	Lack of leadership and lack of internal cohesion, results in an inability to collaborate and lack of strengths-based practice (S-BP)	Leadership is apparent however does not always meet the needs of staff, hindering cohesion, S-BP and collaboration	Consistent leadership begins to foster a culture of shared language, cohesion, S-BP and collaboration	Positive leadership allows staff to work collaboratively and leads to cohesion, S-BP and shared language use most of the time	Positive and supportive internal leadership that actively fosters cohesion, shared language, S-BP and meaningful collaboration	
**2.2 | Shared vision, common direction, culture and purpose**	Professionals within the team have greatly differing priorities and a lack of understanding of what matters to others	Professionals within the team share some priorities but differ on others and have a basic understanding of what matters to others	Professionals within the team largely share priorities. They engage with others to foster their understanding of what matters	Professionals within the team have a comprehensive understanding of what matters for others and work together on key priorities	Professionals within the team share vision and working culture, supporting each other to realise these joint aims and common good	
**2.3 | Opportunities for meeting and discussion**	Limited opportunities to meet internally across professional groups leading to isolated and silo working within the team	Irregular opportunities to meet across professional groups so staff may engage with others, but may still act in isolation	Regular but infrequent opportunities for staff to meet internally across groups – collaboration is sought but is inconsistent	Consistent opportunities for staff to meet internally across groups leading to increased collaboration within the team	Regular meetings internally and with other professionals within the team to discuss and maintain collaborative working	
**2.4 | Networking with others**	Lack of active networking with others – M-PT may only reluctantly collaborate when approached to do so by others	Limited networking between services and staff leading to limited collaboration opportunities	Beginnings of consistent networking between staff members and services to develop connections	Consistent networking attempts between staff members and services that fosters connections and collaboration	Engagement in active networking, forming trusting connections and develops collaboration with others	
**2.5 | Communication between professionals**	Professionals do not communicate outside of their team – service users may need to continually repeat themselves to other teams	Professionals communicate with others when necessary (i.e. in the case of high risk situations) but do not do so regularly	Consideration of establishing routes for communication with other professionals to begin risk sharing and avoid repetition	Communication routes established leading to increased sharing between professionals that effectively manages risk and includes processes for review	Open and clear communication between professionals via established routes – no 'repetition' needed for those that the M-PT is there to support	

Domain	Descriptors: For each of the dimensions below, which statement below (S1-S5) best describes your current position?<br>*It is important to note that there is an underlying logic in how the statements build on one another across the matrix. The statements are broadly incremental – **moving along the boxes presupposes that forms of practice under the previous statement are included in the next one**.*<br>*Darker shading against statements indicates that there is evidence that the statement has been fully achieved. Lighter shading is an indication that some progress has been made in this domain, but that it remains a 'work in progress'.*					
	S1	*S2*	*S3*	*S4*	*S5*	*N/A*
**TEST QUESTIONS FOR DOMAIN 2**<br>(from RLFfP, Q2)	**WHAT IS THE DISTINCTIVE ROLE OF THIS PARTNERSHIP?**<br>▪ How do you see your work leading to the change you want?<br>▪ What other work is going on in your area/system on this topic?<br>▪ How is your work distinct from and related to that work, and what does it contribute?					
**Useful resources and worked examples:**	Addressing the practical challenges of multidisciplinary teamwork in primary care | Nuffield Trust<br>Practice-integrated care teams – learning for a better future | Emerald Insight<br>General practice: case studies of GP organisations working at scale to deliver access and continuity | Nuffield Trust<br>Talking together in rural palliative care: a qualitative study of interprofessional collaboration in Norway | SpringerLink					

Domain	Descriptors: For each of the dimensions below, which statement below (S1-S5) best describes your current position?<br>*It is important to note that there is an underlying logic in how the statements build on one another across the matrix. The statements are broadly incremental – **moving along the boxes presupposes that forms of practice under the previous statement are included in the next one**.*<br>*Darker shading against statements indicates that there is evidence that the statement has been fully achieved. Lighter shading is an indication that some progress has been made in this domain, but that it remains a 'work in progress'.*					
	S1	*S2*	*S3*	*S4*	*S5*	*N/A*
**3. EXTERNAL RELATIONSHIPS** (Link to relevant film on SPPC channel: Domain 3)						
**Most important & impactful data items:** co-ordinated service provision; reduced risk of harm; the right person providing the right support at the right time; delivery of safe, high quality, effective care; trust and relationships between organisations						
**3.1 | Engagement with social services**	No established routes of communication between M-PT and social services – each working in isolation	The M-PT has a means of communication with social services however this may be inconsistent or cumbersome, or too reliant on individuals	The M-PT communicates with social services when necessary and has an established channel to do so	The M-PT and social services are able to communicate with each other but may do so irregularly	Clear, consistent high-quality two-way communication between M-PT and social services leads to effective and efficient ways of working	
**3.2 | Engagement with the third sector**	Lack of understanding of the role of the third sector and no communication established with key partners	Consideration of the role of third sector is undertaken and communication may be established via a third party	The M-PT has a basic understanding of the role of the third sector and has a working relationship with key partners	The M-PT has a good understanding of the importance of the third sector and engages in regular communication	Well established routes for open communication between primary care and third sector partners leading to joined-up, fluid working	
**3.3 | Engagement with secondary care / community hospitals**	Service and key parts of secondary care / community hospitals only communicate when required, and lack understanding of one another	Service and key parts of secondary care / community hospitals communicate irregularly and this may be cumbersome for them – they have a basic understanding of each other	Service and key parts of secondary care / community hospitals establish a route for communication to increase understanding and reduce burden	Service and key parts of secondary care / community hospitals communicate regularly through a consistent route and have a decent understanding of one another	Service and key parts of secondary care / community hospitals communicate effectively through an established route and have an excellent understanding of one another	
**3.4 | Engagement with primary care**	Service and key parts of primary care only communicate when required, and lack understanding of one another	Service and key parts of primary care communicate irregularly and this may be cumbersome for them – they have a basic understanding of each other	Service and key parts of primary care establish a route for communication to increase understanding and reduce burden	Service and key parts of primary care communicate regularly through a consistent route and have a decent understanding of one another	Service and key parts of primary care communicate effectively through an established route and have an excellent understanding of one another	

Domain	Descriptors: For each of the dimensions below, which statement below (S1-S5) best describes your current position?<br>*It is important to note that there is an underlying logic in how the statements build on one another across the matrix. The statements are broadly incremental – **moving along the boxes presupposes that forms of practice under the previous statement are included in the next one**.*<br>*Darker shading against statements indicates that there is evidence that the statement has been fully achieved. Lighter shading is an indication that some progress has been made in this domain, but that it remains a 'work in progress'.*					
	S1	*S2*	*S3*	*S4*	*S5*	*N/A*
**3.5 | Engagement with other key stakeholders** [only if additional and relevant to 3.1, 3.2, 3.3]	Low quality and limited relationships with other important stakeholders	Growing awareness of other stakeholders of the importance and function of the M-PT	Emergence of key contacts and partners who have an understanding of the aim and purpose of the M-PT	Good quality relationships in place, but are not optimised - constraints are still in place around the partners	Effective relationships and networks established in order to further aims of the M-PT	

**TEST QUESTIONS FOR DOMAIN 3**<br>(from RLFfP, Q3)	**WHO ARE THE PARTNERSHIP'S MEMBERS AND STAKEHOLDERS?**<br>▪ How are you learning about the knowledge, skills, perspectives, interests, connections and wider resources that your members bring?<br>▪ Who are your stakeholders, and how are you engaging them in your work?<br>▪ What are your accountabilities and how might that impact on making decisions?<br>▪ What do changes in your membership mean for the range of skills, knowledge, and perspectives, and your capacity to work towards your purpose?<br>▪ How can you create regular spaces in which informal and personal connections can develop among members?
**Useful resources and worked examples:**	Impact of 'Enhanced' Intermediate Care Integrating Acute, Primary and Community Care and the Voluntary Sector in Torbay and South Devon, UK - PMC (nih.gov)<br>What steps are currently being taken to reform social care? | Nuffield Trust<br>Social care providers and integrated care systems: opportunities and challenges | The King's Fund (kingsfund.org.uk)<br>Primary care home and social care: working together (napc.co.uk)

Domain	Descriptors: For each of the dimensions below, which statement below (S1-S5) best describes your current position? *It is important to note that there is an underlying logic in how the statements build on one another across the matrix. The statements are broadly incremental – **moving along the boxes presupposes that forms of practice under the previous statement are included in the next one**.* *Darker shading against statements indicates that there is evidence that the statement has been fully achieved. Lighter shading is an indication that some progress has been made in this domain, but that it remains a 'work in progress'.*					
	S1	*S2*	*S3*	*S4*	*S5*	*N/A*
**4. INFORMATION SHARING AND GOVERNANCE** (Link to relevant film on SPPC channel: Domain 4)						
**Most important & impactful data items:** using shared IT systems, agreed processes that enable seamless delivery between teams and services; one system that supports people by putting them at the centre						
**4.1 | Appropriate IT systems access**	Service does not have the appropriate level of access to relevant IT systems and may indeed lack access altogether	Service has inconsistent level of access to some IT systems and may still lack access to others	Service has some access to all relevant IT systems however may not always have the access required (i.e. read only)	Service has requisite level of access to most relevant IT systems	Service has requisite level of access to all relevant IT systems – which alleviates staff burden	
**4.2 | Communication between IT systems**	IT systems are isolated and unable to communicate with one another leading to high duplication of information	IT systems may have some communication between one another however this may be cumbersome to use and does not reduce duplication	IT systems are usually able to communicate between one another however some duplication is still needed	IT systems communicate effectively however are not inter-operable, and so a small amount of duplication may be needed	IT systems are entirely inter-operable between services and agencies removing the need for duplication	
**4.3 | Record keeping**	Record keeping is sub-optimal leading to questions over whether information and data is gathered effectively	Record keeping is usually consistent however there are irregularities in this between professionals	Record keeping is consistent but basic and does not always provide the required level of detail	Record keeping is consistent and clear however small amounts of data may still be missed when passed to other professionals	Robust and reliable record keeping that allows data to be safely passed between professionals with ease	
**4.4 | Confidentiality and consent**	Lack of clear guidance on confidentiality and consent – people are unaware of how to share information	Limited guidance provided to staff on confidentiality and consent however staff lack confidence in their knowledge of these processes	Basic confidentiality and consent guidance provided to staff who have the confidence to explain this to others	Clear confidentiality and consent procedures that all staff are familiar with and confident in their use	Clear, cohesive guidance on confidentiality and consent between services that professionals have high confidence in, and that secures the safety of people	

Domain	Descriptors: For each of the dimensions below, which statement below (S1-S5) best describes your current position?<br>*It is important to note that there is an underlying logic in how the statements build on one another across the matrix. The statements are broadly incremental – **moving along the boxes presupposes that forms of practice under the previous statement are included in the next one**.*<br>*Darker shading against statements indicates that there is evidence that the statement has been fully achieved. Lighter shading is an indication that some progress has been made in this domain, but that it remains a 'work in progress'.*					
	S1	*S2*	*S3*	*S4*	*S5*	*N/A*
**4.5 | Clear policies and procedures**	Lack of cohesive team policy and procedure leading different professionals taking different actions	Consideration of team policy and procedure to standardise the actions of different professionals	Basic policy and procedure implemented throughout the team however some professionals may lack clarity and still act differently	Good team policy and procedure that all members of the team have some understanding of	Clear M-PT-specific policy and procedure that all team members understand and follow, regardless of employer	
**4.6 | Robust systems for information governance**	Information governance is not given sufficient focus and is a significant barrier to successful M-PW	More attention is paid to the requirements of information governance, but this is very much still work in progress	Information governance has a growing place with the M-PT and is understood by all partners and given increasing attention	Systems and procedures are in place which facilitate good information governance, but room for improvement exists	Information governance is a top priority for the M-PT and is a key enabler of being able to work effectively as a team	

**Useful resources and worked examples:**	A Case Study of Implementing Grant-Funded Integrated Care in a Community Mental Health Center - PMC (nih.gov)<br>Much more to do: Understanding the impact of technology in social care | Nuffield Trust<br>Enablers and Barriers for Collaboration – Introducing Digital Safety Alarms for Elderly Living at Home - International Journal of Integrated Care (ijic.org)

Domain	Descriptors: For each of the dimensions below, which statement below (S1-S5) best describes your current position?<br>*It is important to note that there is an underlying logic in how the statements build on one another across the matrix. The statements are broadly incremental – **moving along the boxes presupposes that forms of practice under the previous statement are included in the next one**.*<br>*Darker shading against statements indicates that there is evidence that the statement has been fully achieved. Lighter shading is an indication that some progress has been made in this domain, but that it remains a 'work in progress'.*					
	S1	*S2*	*S3*	*S4*	*S5*	*N/A*
**5. EQUITY AND EQUALITY** (Link to relevant film on SPPC channel: Domain 5)						
**Most important & impactful data items:** respect for others, supportive team climate, valuing the peoples voice; the right person providing the right support at the right time; a happy workforce having a positive impact on patient outcomes						
**5.1 | Geographical equity**	Unwarranted 'postcode lottery' with regard to opportunities and service provision	Consideration given to equity across areas however service provision is still inconsistent	Beginnings of enacting equity across areas leading to increased consistency in service provision	Service provision and opportunities are broadly equitable across areas, however slight variation is still present	Service provision and opportunities are entirely equitable across different areas	
**5.2 | Managing capacity**	Capacity is improperly managed leaving team members with undue variation of available time	Capacity of staff members is considered and there is a beginning of the management of this	Capacity is managed across the team to an extent, however it is highly variable (e.g. it is 'vulnerable' to annual leave or sickness)	Capacity is usually well managed and fair however slight variation across staff members is present	Team members feel that capacity is managed fairly and that there is equitable allocation across the M-PT, even in the case of absences	
**5.3 | Parity of esteem**	Different roles are treated with differing levels of respect and not trusted or given equal opportunities to develop relationships with others	All roles are shown some respect and are valued to an extent but are not given equitable opportunities to others	All roles are shown equitable respect and value in the majority of cases – opportunities to collaborate are encouraged but are inconsistent across roles	All roles are shown equitable respect and value, though may have some slight differences in their opportunities to collaborate or develop relationships	All roles are respected, valued, and given equal status with the M-PT	
**5.4 | Voice of those closest to the person**	Members of the team who are closest to the person (i.e. HCSW's) may not be listened to or involved in decision making	There is inconsistency in the extent to which members of the team closest to the person are listened to or involved in decision making	Members of the team closest to the person are always listened to however there may be variation in the extent to which their voice is implemented in care	Members of the team closest to the person are always involved and encouraged to partake in discussions about wellbeing needs/outcomes but some variation is present	Members of the team closest to the person are always actively engaged in discussions regarding a person's wellbeing needs and outcomes	

Domain	Descriptors: For each of the dimensions below, which statement below (S1-S5) best describes your current position?<br>*It is important to note that there is an underlying logic in how the statements build on one another across the matrix. The statements are broadly incremental – **moving along the boxes presupposes that forms of practice under the previous statement are included in the next one**.*<br>*Darker shading against statements indicates that there is evidence that the statement has been fully achieved. Lighter shading is an indication that some progress has been made in this domain, but that it remains a 'work in progress'.*					
	S1	*S2*	*S3*	*S4*	*S5*	*N/A*
**Useful resources and worked examples:**	Resilience is much more than hospital beds | The King's Fund (kingsfund.org.uk)<br>To unlock innovation, we need to work with the people who draw on care | The King's Fund (kingsfund.org.uk)<br>Dying well at home: Three things I've learnt about working with patients, carers and families in end-of-life care | The King's Fund (kingsfund.org.uk)					

Domain	Descriptors: For each of the dimensions below, which statement below (S1-S5) best describes your current position?<br>*It is important to note that there is an underlying logic in how the statements build on one another across the matrix. The statements are broadly incremental – **moving along the boxes presupposes that forms of practice under the previous statement are included in the next one**.*<br>*Darker shading against statements indicates that there is evidence that the statement has been fully achieved. Lighter shading is an indication that some progress has been made in this domain, but that it remains a 'work in progress'.*					
	S1	*S2*	*S3*	*S4*	*S5*	*N/A*
**6. PERSON-CENTRED PRACTICE** (Link to relevant film on SPPC channel: Domain 6)						
**Most important & impactful data items:** person receiving timely, coordinated, collaborative care**;** maximising peoples independence; enabling people to have involvement in their care; supporting people to live well; shared decision making with the person; better care and clarity for people and their families; person receiving care in a location and format that best meets their needs; accessible support and care; wanting to do right by the person; measuring service user perspective; more focus on early intervention						
**6.1 | Focus and responsivity of the service**	A 'tick box' service is delivered without sufficient holistic consideration of the person being supported	People's needs are considered on commencement of the service, but M-PT offer may be insufficiently responsive	Service flexibility and responsivity is provided in some, but not all, areas and so does not always address needs holistically (e.g. number / length of appointments)	Service is mostly flexible and responsive however some people may still feel this does not meet their outcomes	Full flexibility and responsivity for the service to be adapted, within established parameters, to a person's outcomes therefore providing a truly holistic service offer	
**6.2 | Flexibility and responsivity of the practitioner**	Professionals only work within rigid guidelines and are not able to provide a flexible and responsive service	Professionals consider how they can start to provide flexible and responsive practice	Professionals provide flexibility and responsivity in some, but not all, areas (i.e. in the groups they signpost)	Professionals are considerate of and responsive to a person's needs and adjust the service offer within the bounds of their knowledge	Professionals are confident and knowledgeable and able to provide a fully flexible and responsive service offer	
**6.3 | Clear definition of professional roles**	Lack of role definition leads to tension / confusion regarding responsibilities within the team and confusion for the person about who will be involved and when	Some roles may have definition however there is limited understanding of this among others	All roles have a definition however there may not be clearly defined boundaries/responsibiliti es established for these	Good definition of professional roles with a good understanding from others of what this entails leading to increased clarity for the person	Clearly defined professional roles and boundaries that all team members understand, respect, trust and value giving the person clarity on who is involved in their care and when	

Domain	Descriptors: For each of the dimensions below, which statement below (S1-S5) best describes your current position? *It is important to note that there is an underlying logic in how the statements build on one another across the matrix. The statements are broadly incremental – **moving along the boxes presupposes that forms of practice under the previous statement are included in the next one**.* *Darker shading against statements indicates that there is evidence that the statement has been fully achieved. Lighter shading is an indication that some progress has been made in this domain, but that it remains a 'work in progress'.*					
	S1	*S2*	*S3*	*S4*	*S5*	*N/A*
**6.4 | Feedback from people supported by the service**	People are given limited opportunity to give their feedback to the M-PT – co-production of the service model is very limited	People have some opportunity to give feedback however they often do not as feedback is not usually implemented or acted upon	People are usually asked to provide service feedback- this is easy to give, sometimes implemented, but there is variation in this	People are always asked to provide service feedback, or contribute to co-production - this is easy to give and usually implemented and acted upon	People are actively encouraged, and want, to provide evidence to actively co-produce the service, which is easy to give, and always acted upon	
**6.5 | Clarifying what matters to the person the service is supporting**	What matters to the person is not given due consideration by the service and people may not be asked as a result of this	People are usually asked about what matters to them however this may not be actively followed up or implemented	People are always asked about what matters to them but there is inconsistency in the extent to which this is followed up or in the quality of these conversations	People are actively encouraged to discuss what matters to them and consistent efforts to integrate this into their care are made	People are actively encouraged to talk about what matters to them including their strengths / wishes / concerns and this is always integrated into their care	
**6.6 | Involvement and communication with a person's support network** <br><u>(where appropriate to the needs of the person)</u>	Communication with a person's family, carer(s) or wider support network is only undertaken when requested by the person themselves	Communication with a person's support network is usually undertaken, but may be inconsistent or limited in scope	Communication with a person's support network is always undertaken and there is increasing depth to these conversations (i.e. discussing what matters)	A person's wider support network is always involved in conversations regarding 'what matters', though there may be some key points missing from discussion	The person's family, carer(s) or wider support network are actively and regularly involved and informed in 'what matters' conversations including discussion of strengths / wishes / concerns	

Domain	Descriptors: For each of the dimensions below, which statement below (S1-S5) best describes your current position? *It is important to note that there is an underlying logic in how the statements build on one another across the matrix. The statements are broadly incremental – **moving along the boxes presupposes that forms of practice under the previous statement are included in the next one**.* *Darker shading against statements indicates that there is evidence that the statement has been fully achieved. Lighter shading is an indication that some progress has been made in this domain, but that it remains a 'work in progress'.*					
	S1	*S2*	*S3*	*S4*	*S5*	*N/A*
**6.7 | Communication and language preferences**	The communication approach of the M-PT is determined by those employed to run it, paying little regard to the communication and language preferences of people	People may be asked about their communication and language preferences however their preference is not often implemented	People are always asked about their preferred communication and language but there is variation in the implementation of this	People's communication and language preferences are always considered and acted upon in the majority of cases	People are able to converse fluently in their language of choice with practitioners, enabling their care and support to be truly person-centred	
**6.8 | Flows through the whole system**	Services lack understanding of each other, do not speak the same 'language', and can be competitive leading to people 'bouncing' between them and needing to repeat their story	Services consider how they can better understand others in order to reduce people 'bouncing' between them and repetition	Services communicate in order to understand one other, begin to share language, and discuss who is the 'right' service at the 'right' time for the person	Services have a good understanding of one another and work towards a shared language – reduction in people 'bouncing' between services and needing to repeat themselves	Services have a collaborative understanding of need, share a common language, and work together to ensure the right people access the right service in the first instance	

TEST QUESTIONS FOR DOMAINS 4, 5 AND 6

(from RLFfP, Q4)

HOW IS WORKING BEING SHARED AND RECOGNISED?

- How will you come together as a partnership in ways that take account of everyone's capacities and preferences?
- Do you have a lead, and what is their role? Has this been agreed collectively? And is it congruent with your values as a partnership?
- How will you communicate between meetings? Which other modes could be useful?
- How will you ensure that power differentials between members are recognised and all voices are equally heard in conversations?
- Do smaller voluntary, community and social enterprise organisations need to be reciprocated for their time? What are the different forms this could take?
- How will you hold yourselves to account for whether you are working in the ways you have agreed?

Domain	Descriptors: For each of the dimensions below, which statement below (S1-S5) best describes your current position?<br>*It is important to note that there is an underlying logic in how the statements build on one another across the matrix. The statements are broadly incremental – **moving along the boxes presupposes that forms of practice under the previous statement are included in the next one**.*<br>*Darker shading against statements indicates that there is evidence that the statement has been fully achieved. Lighter shading is an indication that some progress has been made in this domain, but that it remains a 'work in progress'.*					
	S1	*S2*	*S3*	*S4*	*S5*	*N/A*
**Useful resources and worked examples:**	Top tips for implementing a collaborative commissioning approach to Home First | Local Government Association<br>Digital primary care: Improving access for all? (nuffieldtrust.org.uk)<br>The importance of continuity: four personal viewpoints | Nuffield Trust<br>How to improve continuity in general practice? | Nuffield Trust<br>Psychological Therapies - Public Health Wales (nhs.wales)<br>All Wales Dementia Care Pathway of Standards – enabling improvements in the provision of dementia care for individuals and their carers | Improvement Cymru<br>Annual Health Checks - Resource Pack for Delivering Health Care to People with a Learning Disability | Improvement Cymru<br>The Health Profile - for adults - Public Health Wales (nhs.wales)<br>The Health Profile - for children and young people - Public Health Wales (nhs.wales)<br>Dementia Friendly Hospital Charter for Wales | Public Health Wales					

Domain	**Descriptors: For each of the dimensions below, which statement below (S1-S5) best describes your current position?**<br>*It is important to note that there is an underlying logic in how the statements build on one another across the matrix. The statements are broadly incremental – **moving along the boxes presupposes that forms of practice under the previous statement are included in the next one**.*<br>*Darker shading against statements indicates that there is evidence that the statement has been fully achieved. Lighter shading is an indication that some progress has been made in this domain, but that it remains a 'work in progress'.*					
	S1	*S2*	*S3*	*S4*	*S5*	*N/A*
**7. RESOURCES – HUMAN AND FINANCIAL** (Link to relevant film on SPPC channel: Domain 7)						
**Most important & impactful data items:** staff wellbeing; staff satisfaction; a happy workforce having a positive impact on patient outcomes; staff retention; empowering staff to work more effectively						
**7.1 | Staff retention**	High staff turnover and the use of agency/locums creates difficulty in maintaining multi-professional relationships	Moderate turnover of staff meaning a high use of agency/locums is still necessary, though some staff may be able to form relationships with others	Variation in staff turnover (i.e. some roles have better retention than others) leading to variability in ability to form multi-professional relationships	Generally low staff turnover that fosters positive multi-professional relationships and keeps the use of agency/locums minimal	Low staff turnover and high retention leading to sustained, and trusting multi-professional relationships	
**7.2 | Staff recruitment**	High number of staff vacancies that places significant additional pressure on existing staff (e.g. increased caseloads)	Moderate number of staff vacancies that places additional pressure on existing staff	Staff vacancies are present however the burden placed on existing staff is manageable until these are recruited to	Staff vacancies are usually kept minimal or recruited to in a timely manner so any additional staff burden is short lived	All staff vacancies are recruited to so that the service can run at capacity sharing the burden across the M-PT	
**7.3 | Budget**	Insufficient budget allocation for M-PT and no budgetary allocation to facilitate multi-professional working (e.g. to allow for protected time)	Minimum budget allocation for sustainable service functioning and very limited budgetary allocation to facilitate M-PW	Budgetary allocation is sufficient for the M-PT to function above its minimum requirements and provide some budgetary allocation for M-PW	Budgetary allocation is sufficient for good service functioning including protected budget to facilitate M-PW	Sufficient, long term sustainable budget that allows for 'headroom' and supports M-PW	
**7.4 | Development of individual team**	Lack of development within the team leading to stagnation of service, and threats to M-PW and a lack of S-BP	Team development is considered however may not be acted upon at this stage	Some team development is implemented though this may not be consistent, and may still hinder M-PW and S-BP	Consistent team development that allows the service to progress and is a facilitator to M-PW and S-BP	Proactive and consistent team development that fosters service innovation, leading to appropriate multi-professional skill mix	

**Domain**	**Descriptors: For each of the dimensions below, which statement below (S1-S5) best describes your current position?**<br>*It is important to note that there is an underlying logic in how the statements build on one another across the matrix. The statements are broadly incremental – **moving along the boxes presupposes that forms of practice under the previous statement are included in the next one**.*<br>*Darker shading against statements indicates that there is evidence that the statement has been fully achieved. Lighter shading is an indication that some progress has been made in this domain, but that it remains a 'work in progress'.*					
	S1	*S2*	*S3*	*S4*	*S5*	*N/A*
**7.5 | Individual staff development**	Limited encouragement for professional development and may not be able to progress where wanted or develop their S-BP	Staff have limited opportunities to develop, may need to organise opportunities themselves and do not have any protected time for it	Staff development is considered for those who want it however their development / learning opportunities may be decided without their input	Staff have input into the opportunities they feel are importance and would enhance their role or foster progression – they have limited protect time for this	Established professional development and progression opportunities provided that encourage S-BP – staff are willing and able to engage and have protected time for this	
**7.6 | Space for physical co-location**	Where co-location is recommended/required, there is very limited or no space for this to be achieved	Co-location is available for some members of staff but not others, or is available inconsistently	Co-location is usually possible for most staff though may need advanced planning (e.g. a rota)	Co-location can be achieved for all staff however some staff may be 'crowded' or lack their own space	Co-location can be achieved with ease where needed, all staff have sufficient space for in person, co-located working	
**7.7 | Virtual co-location**	Poor quality online working arrangements which act as a barrier to M-PW	Online working arrangements are in place but are inconsistent or inoperable for some staff	Online working arrangements work for most staff and allow for some level of M-PW	All staff have access to online working arrangements and are able to use this to facilitate M-PW	Effective and efficient working online which enables and enhances relationship development	

**Useful resources and worked examples:**	Fronting up to the problems: what can be done to improve the wellbeing of NHS staff? | Nuffield Trust<br>New horizons: What can England learn from the professionalisation of care workers in other countries? | Nuffield Trust<br>Developing the digital skills of the social care workforce | Nuffield Trust<br>Co-location, an enabler for service integration? Lessons from an evaluation of integrated community care teams in East London - Wiley Online Library<br>Cloud-based integrated socio-sanitary care e-services in Croatia: Lessons learned (shs-conferences.org)

Domain	Descriptors: For each of the dimensions below, which statement below (S1-S5) best describes your current position?<br>*It is important to note that there is an underlying logic in how the statements build on one another across the matrix. The statements are broadly incremental – **moving along the boxes presupposes that forms of practice under the previous statement are included in the next one**.*<br>*Darker shading against statements indicates that there is evidence that the statement has been fully achieved. Lighter shading is an indication that some progress has been made in this domain, but that it remains a 'work in progress'.*					
	S1	*S2*	*S3*	*S4*	*S5*	*N/A*
**8. EMBEDDING DATA IN PRACTICE** (Link to relevant film on SPPC channel: Domain 8)						
**Most important & impactful data items:** measuring service user perspective; delivery of safe, high quality, effective care; valuing the peoples voice; trust and relationships between organisations; using shared IT systems; reduced risk of harm						
**8.1 | Culture of quality improvement and learning**	Minimal understanding of available systems for use in QI and learning. Practitioners focus on delivery without due consideration of service development	Staff have some understanding of need to engage stakeholders in QI	Service lead undertakes QI/learning due to lack of training in other staff members	Evidence base is developed and presented as part of QI suggestions<br><br>All staff members are able to contribute to QI on some level	Well established relationships with stakeholders for quality improvement from the earliest possible opportunity. All staff have an involvement in the QI being undertaken	
**8.2 | Appropriate range of skills and systems**	Some skills for quality improvement that vary between practitioners. Limited staff appreciation of the value of research/project management skills	Staff may understand the value of skills, but not want to personally contribute	Consideration of how to develop staff research skills and understanding (i.e. by providing training)	Provision of a staff member able to effectively interpret, collect and deploy relevant data. Appropriate levels of research skills embedded across the team at all levels	Practitioners of all levels are able to contribute to research and understand its value. Specific staff member in post to champion research and quality improvement as early as possible	
**8.3 | Quality of data and evidence**	Quality of data gathered / evidence base (e.g. accuracy and consistency) is poor, and typically derived from secondary sources	Quality of data and evidence base is variable across different parts of the service and is not used for service delivery	Quality of data and evidence base is fair and consistent across the service, not all staff have an understanding of this and it is not always used in service delivery	Good quality of data and evidence is gathered and understood by staff and usually informs delivery	Ability to collect consistent, robust data from the service itself which is understood by staff through established feedback loops and informs core delivery	

Domain	Descriptors: For each of the dimensions below, which statement below (S1-S5) best describes your current position? *It is important to note that there is an underlying logic in how the statements build on one another across the matrix. The statements are broadly incremental – **moving along the boxes presupposes that forms of practice under the previous statement are included in the next one**.* *Darker shading against statements indicates that there is evidence that the statement has been fully achieved. Lighter shading is an indication that some progress has been made in this domain, but that it remains a 'work in progress'.*					
	S1	*S2*	*S3*	*S4*	*S5*	*N/A*
**8.4 | Embedding data in processes of improvement**	Data collection is limited and when carried out is not further utilised – little is learned from service data	Data collection is variable across the service and is not usually meaningfully utilised	Data collection is consistent but basic and is not necessarily detailed enough to provide meaningful insights for utilisation	Data collection is consistent and detailed enough to be meaningful. It is usually further utilised however there is slight variation in this	Changes to the service are implemented based on the data gathered. Relevant and rich data is always collected and further utilised in auditing, research or service feedback leading to effective quality improvement	
**8.5 | Shared learning between teams**	Teams develop and learn in isolation and may not share their learning with others, and consequently not improve	Teams develop and learn in isolation however may share this with others for further improvement	Teams begin to develop their learning together and usually share this between one another	Teams consistently learn together and share knowledge however may not use this to inform their services in tandem with one another	Collaborative learning and knowledge sharing between teams from 'day one' allowing them to develop in tandem, and inform core service planning and delivery	
**8.6 | Co-production of service and quality improvement**	Limited interaction with those who use the service, and lack of opportunities to co-produce solutions	Some opportunities for co-production are present however this is usually at a basic level that may not impact service delivery	Co-production is sometimes undertaken and responded to, however there is variation in this across the M-PT	Co-production is consistently undertaken and valued, however the service may not be immediately responsive	Effective mechanisms for learning from others and co-producing a more effective response to service challenges	

**TEST QUESTIONS FOR DOMAINS 7 AND 8** (from RLFfP, Q5)	**HOW ARE YOU LEARNING AND ADAPTING?**<br>▪ How are you balancing your attention between task delivery, how you are working together, and what you are learning?<br>▪ What spaces and times do you have to reflect on your membership, purpose and ways of working?<br>▪ How are you holding yourselves to account for acting on what you are learning?<br>▪ How are you sharing learning in your wider networks?

Domain	Descriptors: For each of the dimensions below, which statement below (S1-S5) best describes your current position?<br>*It is important to note that there is an underlying logic in how the statements build on one another across the matrix. The statements are broadly incremental – **moving along the boxes presupposes that forms of practice under the previous statement are included in the next one**.*<br>*Darker shading against statements indicates that there is evidence that the statement has been fully achieved. Lighter shading is an indication that some progress has been made in this domain, but that it remains a 'work in progress'.*					
	S1	*S2*	*S3*	*S4*	*S5*	*N/A*
**Useful resources and worked examples:**	Enhancing care transfers from hospital to home for older people with complex needs - International Journal of Integrated Care (ijic.org)<br>An Overview of Reviews on Interprofessional Collaboration in Primary Care: Barriers and Facilitators - PMC (nih.gov)<br>Outcome Measures - Public Health Wales (nhs.wales)					
